# TIDD: tool-independent and data-dependent machine learning for peptide identification

**DOI:** 10.1186/s12859-022-04640-y

**Published:** 2022-03-30

**Authors:** Honglan Li, Seungjin Na, Kyu-Baek Hwang, Eunok Paek

**Affiliations:** 1 Department of Computer Science, Hanyang University, Seoul, 04763 Republic of Korea; 2 Institute for Artificial Intelligence Research, Hanyang University, Seoul, 04763 Republic of Korea; 3 School of Computer Science and Engineering, Soongsil University, Seoul, 06978 Republic of Korea

**Keywords:** Mass spectrometry, Peptide identification, PSM rescoring, Tool-independent, Data-dependent, Machine learning

## Abstract

**Background:**

In shotgun proteomics, database search engines have been developed to assign peptides to tandem mass (MS/MS) spectra and at the same time post-processing (or rescoring) approaches over the search results have been proposed to increase the number of confident peptide identifications. The most popular post-processing approaches such as Percolator and PeptideProphet have improved rates of peptide identifications by combining multiple scores from database search engines while applying machine learning techniques. Existing post-processing approaches, however, are limited when dealing with results from new search engines because their features for machine learning must be optimized specifically for each search engine.

**Results:**

We propose a universal post-processing tool, called TIDD, which supports confident peptide identifications regardless of the search engine adopted. TIDD can work for any (including newly developed) search engines because it calculates universal features that assess peptide-spectrum match quality while it allows additional features provided by search engines (or users) as well. Even though it relies on universal features independent of search tools, TIDD showed similar or better performance than Percolator in terms of peptide identification. TIDD identified 10.23–38.95% more PSMs than target-decoy estimation for MSFragger, which is not supported by Percolator. TIDD offers an easy-to-use simple graphical user interface for user convenience.

**Conclusions:**

TIDD successfully eliminated the requirement for an optimal feature engineering per database search tool, and thus, can be applied directly to any database search results including newly developed ones.

**Supplementary Information:**

The online version contains supplementary material available at 10.1186/s12859-022-04640-y.

## Introduction

In shotgun proteomics, peptide-spectrum match (PSM) rescoring is a process of evaluating confidence of PSMs obtained through database search. Database search uses similarities between tandem mass (MS/MS) spectra produced by a mass spectrometry (MS) instrument and theoretical spectra of peptides in the sequence database, as a measure [1]. However, the peptide assignments are often incorrect because MS/MS spectra are deficient due to noise or missing ion peaks [2] and also because MS/MS spectra are sometimes generated from non-peptide species and modified peptides not present in the search database. It has been a critical step to evaluate whether the peptide assignments to spectra are correct.

In the last 20 years, various PSM rescoring algorithms have been developed for confident peptide identification, such as heuristic based H-score [3] and tailor [4], probability model-based Peppy [5], and machine learning (ML)-based Scavanger [6], PeptideProphet [7], Percolator-related tools [8–12] and Qranker [13]. The ML-based PSM rescoring methods can be grouped according to their tool-dependency and data-dependency. The rescoring algorithms are considered *tool-dependent*, if they use tool-specific optimal feature sets for model learning. *Tool-independent* models use universal feature sets regardless of the database search tools of choice. On the other hand, we call the tools *data-dependent* when the ML based models for PSM rescoring are dynamically learned from individual input search results. If the ML models were trained in advance and fixed (i.e., not trained over an individual dataset), we call them *data-independent*.

According to such classification, PeptideProphet [2, 7, 14, 15], the first ML based PSM rescoring method, is tool-dependent and semi data-independent. It calculates a discriminant score for each PSM based on pre-learned parameters, and fits target-decoy score distribution to a mixture model by Expectation–Maximization (EM) algorithm [16]. From the fitted mixture model, a probability that a PSM is correct is assigned to each PSM. We call it tool-dependent because the discriminant score was calculated using tool-specific features and pre-learned coefficients (weights). For instance, the features for Comet search results contained XCorr, delta Cn, Sp score, and Sp rank, which were Comet-specific features and were not adopted when applying PeptideProphet to other database search tools. In addition, the distribution of discriminant scores is fitted to different mixture models depending on the database search tool. For instance, a normal-gamma mixture model is assumed for Comet, and a normal-gumbel mixture model is hypothesized for MSFragger. As for data-dependency, we define the PeptideProphet as semi data-independent. In some sense, it is data-independent because discriminant scores are calculated using pre-learned weights and the hypotheses for mixture models remain the same for given database search tools. However, PeptideProphet dynamically learns the parameters of mixture model from an individual input dataset, so it can also be considered data-dependent at the same time. The pre-learned and pre-determined discriminant score calculation or the mixture model hypotheses may not properly capture the characteristics of each MS/MS dataset, which can be affected by external factors such as instruments, fragmentation methods, and collision energy.

The second group adopts tool-dependent and data-dependent models to overcome the bias of pre-trained models. The tools in this group are gradient boosting-based Scavager [6], and support vector machine-based Percolator-related tools (Percolator [8], MS-GF + Percolator [12], Mascot Percolator [17], X!Tandem Percolator [10, 11], OMSSA Percolator [9], speed-up version of Percolator [18]) and Qranker [13]. Among them, the most widely used tool is Percolator. To eliminate the potential bias caused by various external factors, Percolator learns a data-dependent model for each input experiment: a linear SVM [19] model is trained iteratively. The resulting SVM model outputs a new score for each PSM by combing various PSM features, together with the original scores provided by the search tool. Percolator constructed a data-dependent model by utilizing labels from the target-decoy search [20] (TD), while the learning was conducted with target PSMs identified at 1% FDR (false discovery rate) as a positive training set and decoy PSMs as negative. For the initial training set, FDR is estimated based on the match score provided by the database search tool. From the second iteration on, FDR is estimated using the new PSM score calculated by the learned SVM model from the previous iteration. In order to yield a stable PSM identification set containing a maximal number of true positive hits, Percolator runs up to ten iterations by default. Though it yields the significant improvement in the number of peptide identifications by generating a data-dependent model for each experiment, applicable database search tools have been limited because Percolator used different features depending on database search tools.

Here, we propose a new PSM rescoring tool, called TIDD (Tool-Independent and Data-Dependent PSM rescoring), that can be applied to validate any database search results. For tool-independence, TIDD calculates 25 universal features to characterize PSMs resulting from any database search. For data-dependence, it performs learning and prediction based on the iterative SVM training in the same way as Percolator. TIDD performance was evaluated using two types of database search. The first type is a standard search with a few number of variable modifications, by three publicly available tools, Comet [21], MS-GF +  [22], and MSFragger [23]. Search results of 11 cell line datasets and high throughput HEK293 dataset were compared. When compared with Percolator, TIDD gave the increase in the number of identifications by 6.36% for Comet search results of the 12 human cell line datasets. In addition, TIDD identified 16.13–38.95% more PSMs than target-decoy estimation for MSFragger search results. The second type is a modification search. In the analysis of the phosphorylation enriched dataset using Comet, TIDD resulted in 13.68% and 2.79% improvement compared to TD and Percolator respectively. When TIDD was applied to the MODplus result searched with 946 variable modifications, TIDD also identified 10.23% and 2.05% more PSMs than TD and the validation results supported by MODplus respectively. We showed that TIDD performance was comparable to or better than those of existing rescoring tools without using any tool-specific match scores while TIDD could be applied to validate database search results. TIDD does not ensure the optimal validation of open mass search results and all the analyses in this study were conducted for closed searches using a tight mass tolerance for precursor ions. We also want to emphasize a special function of TIDD that allows users to directly add PSM features for rescoring, i.e., users can develop their own rescoring models for any search tool. TIDD offers graphical user interfaces for easy access to such functions.

## Experimental procedures

### Human cell line data sets

We used two types of human cell line data sets. The first type is global profiling datasets—11 human cell line datasets and HEK293 dataset. 11 human cell line data sets (PRIDE ID: PXD002395) was studied by Geiger and colleagues, acquired using an LTQ-Orbitrap Velos mass spectrometer (Thermo Fisher Scientific) coupled with high performance liquid chromatography (HPLC). The MS/MS scans obtained from 11 human cell lysates—A549, HEK293, GAMG, HeLa, HepG2, Jurkat, K562, MCF7, RKO, and U2OS cells, were composed of 136,309, 148,800, 152,777, 159,455, 149,974, 160,225, 167,429, 174,709, 164,317, 161,334, and 165,271 scans respectively. The second data set was high-throughput HEK293 data, composed of 1,121,149 scans generated by a Q-Exactive Orbitrap mass spectrometer [24] (PRIDE ID: PXD001468). The second type is the phosphorylation enrichment dataset. We used the human epithelial cervix carcinoma Hela cells (female), which was studied by Bekker-Jensen and colleagues [25]. The Hela phosphorylation data were analyzed on an EASY-nLC 1000 coupled to a Q-Exactive HF instrument (Thermo Fisher Scientific), coupled with a high pH reversed-phase HPLC fraction. The number of MS/MS spectra obtained from the Hela dataset was 362,356.

### Standard search

All the MS/MS spectra were searched against a target-decoy protein database, which consisted of 42,258 SwissProt human protein (ver. 2017/12) sequences, 179 common contaminants and their pseudo-reversed sequences. To see the PSM rescoring effect on database search tools, we searched MS/MS spectra using 3 database search tools—Comet (v2017013) [21], MS-GF + (v9969) [22], and MSFragger (v20171106) [23] with the following parameters. 20 ppm (or 5 ppm) precursor mass tolerance, 0.02 (or 0.01) fragment mass bin tolerance, ^13^C isotope error, and up to 2 missed cleavage sites, one fixed modification (Carbamidomethyl at Cys) and one variable modifications (Oxidation at Met) were set for 11 cell line (or HEK293) Comet search. For the MS-GF +, “-t 20 ppm -ti -1,2 -tda 0 -m 3 -inst 1 -e 1 -tt 1 -addFeatures 1” and “-t 5 ppm -ti -1,2 -tda 0 -m 3 -inst 1 -e 1 -tt 1 -addFeatures 1” were used for 11 cell line and HEK293 dataset respectively, with the same modification settings as Comet. For MSFragger, we did a closed search with the parameter set—precursor mass tolerance = 20 ppm, precursor true tolerance = 20 ppm, fragment mass tolerance = 0.025 Da, isotope error = 2, num_enzyme_termini = 1, allowed_missed_cleavage = 2, Carbamidomethl at Cys, and Oxidation at Met modification on 11 cell line dataset. For the HEK293 MSFragger search, we changed precursor mass tolerance, precursor true tolerance values to 5 ppm. After the database search, we ran Percolator (ver 3.02.0).

### Modification search

Two types of modification search were conducted. One is phosphorylation search on the Hela phosphorylation enrichment dataset. 362,356 MS/MS spectra were searched by Comet against a Uniprot database (v211103), which consists of a total 100, 279 protein sequences, including 182 contaminants. For Comet search, a set of parameters used was as follows: peptide mass tolerance=20ppm, isotope error=2, search_enzyme_number=2, fully digested, one fixed Carbamidomethyl at Cys, two variable modifications (Oxidation at Met and Phosphorylation at Ser, Thr, and Tyr). After the Comet search, we ran Percolator (ver 3.02.0). The other search is for modification search on HEK293 dataset. MODplus search was conducted with exactly the same parameters as in S. Na et al. [26]: ^13^C isotope errors of − 1, 0, +1, and +2 in precursor ions, fully/partially tryptic peptides of arbitrary number of missed cleavages, and any number of modifications per peptide. The number of considered variable modifications were 946 (Unimod, v201807), whose masses were restricted between − 150 and +350 Da. For the database, the Uniprot human reference proteome (v201806) was used, which consists of 93,793 proteins.

### TIDD PSM rescoring method

**Fig. 1:**
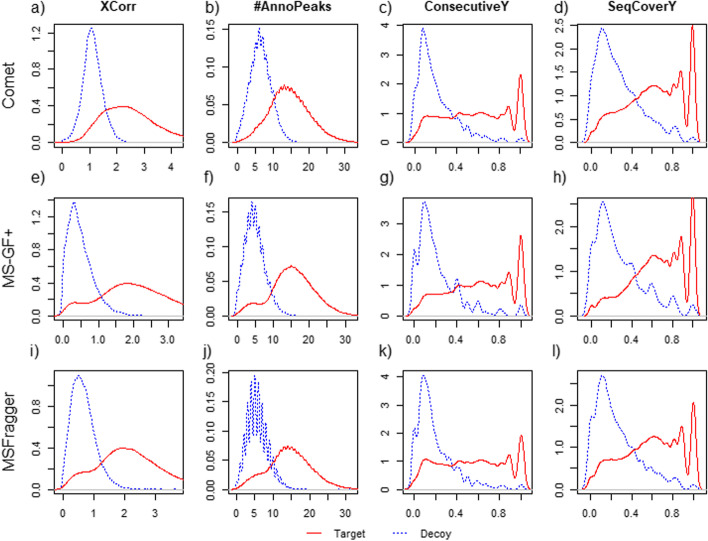
The target and decoy distributions of TIDD’s top 4 features. The results are based on A549 dataset searched by **a**–**d** Comet; **e**–**h** MS-GF + ; **i**–**l** MSFragger. Here, solid and dashed line are the distribution of target and decoy, respectively

The essential part of the TIDD model is the use of tool-independent features regardless of database search tools. To eliminate tool dependency, we use the features about fragment ion annotation and cross-correlation score (XCorr) as a PSM score. Note that we have directly calculated XCorr as a universal score of a PSM from any search tool. The TIDD features, shown in Table 1, can largely be categorized into three types: the features about (1) basic PSM information, (2) fragment ion annotation, (3) and the overall quality of peptide and spectrum match. We confirmed the discriminatory power of these features using the target and decoy hits from A549 dataset, one of the 11 cell line datasets. The distributions of the top 4 features are shown in Fig. 1 (The remaining 20 features’ distributions are shown in Additional file 1: Figures S1 to S3). The best distinguishing feature between target and decoy hits was XCorr. Though the distributions of these four features are slightly different depending on the search tool, it clearly distinguishes target and decoy distributions regardless of database search tools.

**Table 1:** Features used to represent PSMs in TIDD model

Index	Name	Description
1	XCorr	cross correlation between theoretical and experimental spectra
2	delta XCorr	difference of XCorr score between rank 1 and 2 (If there’s rank 2 hit)
3	charge	vector: 1 to 6 (consider as 6 when the charge is above 6)
4	pepLen	the length of stripped peptide sequence
5	tryptic	vector: 0 c-term tryptic; 1 n-term tryptic; 2 fully-tryptic
6	#missed cleavage	the number of missed cleavages in the peptide sequence
7	precursorM	observed mass of spectra
8	massDiff	the mass difference between calculated and observed mass
9	-absolutMassDiff	the absolute value of the difference between calculated and observed mass
10	calPepM	calculated mass of the matched peptide
11–13	sum_intensity_all/y/b	logarithm value of sum of intensity of spectra (TIC) / sum of intensity of matched y ions (or b ions)
14–15	frac_intensity_y/b	the fraction of sum_intensity_y (sum_intensity_b) among sum_intensity_all
16–18	max_intensity_all/y/b	logarithm value of maximum intensity of spectra (base peak intensity) / maximum intensity of matched y ions (or b ions)
19–20	seq_cover y/b	sequence coverage of y ions (or b ions)
21–22	num_consecutive_y/b	the number of consecutive y ions (or b ions)
23–24	mean/sd _fragMassErr	mean (or standard deviation) values of mass difference distribution between fragment ions and theoretical ions
25	#AnnoPeaks	the number of annotated peaks

Based on these features, we trained SVM models iteratively as Percolator. The target PSMs identified at XCorr-based FDR 1% are used as a positive training set during the 1st iteration of SVM learning. From the 2nd iteration on, the learned SVM scores were used to estimate FDR and choose positive training instances. As for the number of positive and negative training instances, we randomly chose 10,000 target and 10,000 decoy PSMs as positive and negative training sets, respectively. This is based on the previous report [27], which showed that SVM performance did not significantly degrade even if only 0.1% of the whole PSMs were used for learning SVM models in large scale proteomics experiments containing about 1 million scans.

To evaluate the TIDD performance, we compared TIDD with target-decoy (TD) approach and Percolator. The PSMs were identified at 1% FDR using e-value scores for TD, q-values for Percolator, and learned SVM scores for TIDD.

## Results

### TIDD performance over standard search of 11 cell line datasets

**Fig. 2:**
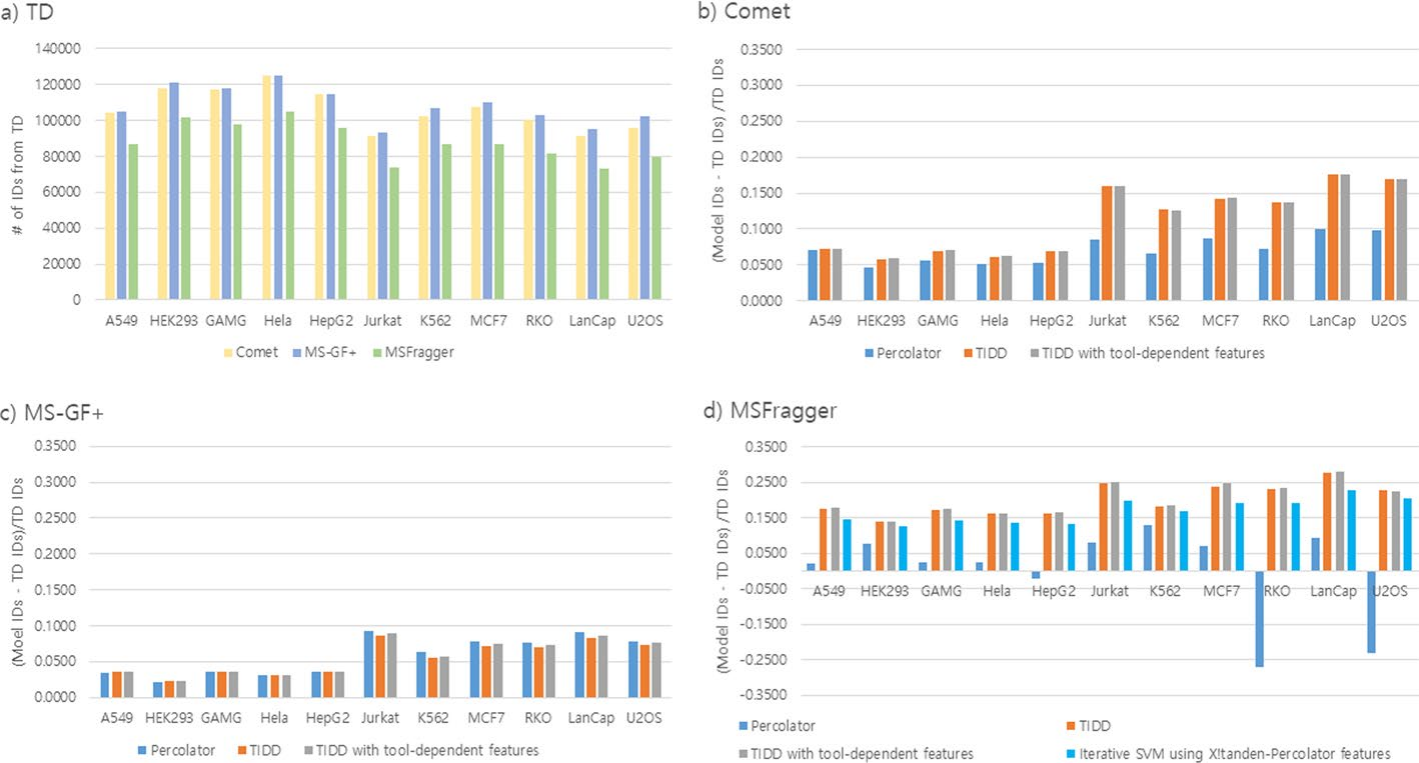
Performance comparison in terms of PSM identifications using 11 cell line datasets. Four PSM rescoring methods were applied—Percolator, TIDD (iterative SVM learning with tool-independent feature set), TIDD with tool-dependent features (iterative SVM learning with TIDD features augmented with tool-dependent scores), and the iterative SVM using X!Tandem-Percolator features (iterative SVM learning with the feature set used by Percolator on X!Tandem data, while ‘deltascore’ is missed because MSFragger does not provide this score). **a** the number of identified PSMs based on TD when the three tools were applied; **b**–**d** the percent increase in PSM identification numbers compared to TD results

The performance of TIDD was compared with those of TD and Percolator. For 11 cell line datasets, TD identified 106,198 ± 11,268 for Comet, 108,436 ± 11,833 for MS-GF +, and 88,101 ± 10,728 PSMs for MSFragger at 1% FDR (Fig. 2a). The numbers of PSMs identified by TD were used as a base line to evaluate the performance of Percolator and TIDD. Figure 2b–d shows how much Percolator and TIDD rescoring improved the number of PSM identifications for each search tool when compared with TD for each 11 cell line data set.

For Comet search results (the detailed numbers are shown in Additional file 1: Table S1), TIDD identified 11.26% ± 4.70% more PSMs than TD, while Percolator identified 7.13% ± 1.90% more PSMs than TD (Fig. 2b). TIDD identified consistently more PSMs than Percolator (Fig. 2b), showing improvements by 0.09% in A549 dataset and 6.91% in Jurkat dataset. The identified PSMs based on TIDD included over 99.20% of Percolator results in each dataset. For MS-GF + search results, the performance of TIDD is shown in Fig. 2c and Additional file 1: Table S2. TIDD identified 2.27% to 8.56% more PSMs than TD, but -0.33 ± 0.38% less PSMs when compared with Percolator. Among the three search tools, MS-GF + identified the highest number of PSMs by TD and thus the improvement was less significant, showing the effectiveness of its e-value score alone in distinguishing correct and incorrect PSMs.

For MSFragger search results, TIDD showed considerable improvements for all 11 cell line datasets over TD and identified 20.12 ± 4.39% more PSMs as shown in Fig. 2d. Because Percolator does not provide an optimized feature set for MSFragger, we implemented MSFragger-Percolator as iterative SVM model learning using the X!Tandem-Percolator feature set instead, because MSFragger adopted X!Tandem algorithm. Compared to the model, TIDD identified 2.72 ± 1.09% more PSMs.

Unlike Comet and MSFragger results, the difference between TIDD and Percolator is less than 1% for MS-GF + results. It is understandable because TIDD uses the general feature set that can be applied to any database search tool while Percolator feature sets are optimized for a specific database search tool.

To see the effect of tool-dependent features, we additionally included the tool-specific scores on top of TIDD features and the performance results were shown as “TIDD with tool-dependent features” in Fig. 2. For Comet, “deltacn”, “deltacnstar”, “SpRank”, logarithm value of “e-value” were added. For MS-GF +, “denovo score”, “MSGF score”, “spectrum e-value”, “e-value” were added and “hyper score”, “next score” and “e-value” were additionally included for MSFragger. The use of tool-specific scores in addition to TIDD features have little effect on the number of PSMs, showing the increase of 0.05 ± 0.07%, 0.13 ± 0.05%, and 0.15 ± 0.22% over TIDD for Comet, MS-GF + and MSFragger, respectively.

### TIDD performance over standard search in large scale dataset

**Fig. 3:**
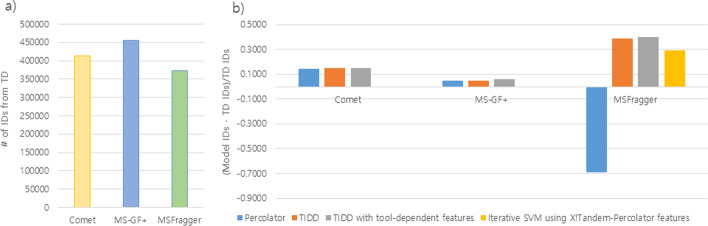
Performance comparison on PSM identification for HEK data. Four PSM rescoring methods were applied—Percolator, TIDD (iterative SVM learning with tool-independent feature set), TIDD with tool-dependent features (iterative SVM learning with TIDD features augmented with tool-dependent scores), and the iterative SVM using X!Tandem-Percolator features (iterative SVM learning with the feature set used by Percolator on X!Tandem data, while ‘deltascore’ is missed because MSFragger does not provide this score). **a** The number of identified PSMs based on TD when the three tools were applied; **b** the percent increase in PSM identification numbers compared to TD results

To assess the performance of TIDD for high throughput proteomics experiments from different MS instruments, we applied TIDD to HEK293 dataset of ~ 1 million MS/MS scans. Figure 3 shows that TD estimation identified 413,367, 456,073, and 372,231 PSMs for Comet, MS-GF +, and MSFragger at 1% FDR, respectively. For Comet search results, TIDD showed 14.72% and 0.24% improvements over TD and Percolator, respectively. For MS-GF + results, TIDD showed 4.72% increase and 0.33% decrease in the number of PSM identifications when compared to TD and Percolator, respectively. For MSFragger, TIDD identified 38.95% and 7.56% more PSMs than TD and the iterative model with X!Tandem-Percolator features, respectively. In addition, when we included tool-dependent features on top of TIDD features, the performance always showed improvements over TD and Percolator regardless of search tools.

### TIDD performance over modification search

To evaluate the TIDD performance in modification search, we applied TIDD to validate the search results of a phosphorylation enrichment of Hela data set. As shown in Table 2, TD estimation identified 152,186 PSMs at 1% FDR and Percolator identified 167,925 PSMs. TIDD identified 172,742 PSMs, which improved the identification performance by 13.51% and 2.79% for TD and Percolator, respectively. We also applied TIDD to validate the results from unrestrictive modification search, where HEK293 data set was searched by MODplus considering 946 variable modifications. The FDR module provided by MODplus identified 653,660 PSMs at 1% FDR, including 216,858 modified PSMs. TIDD worked well for such modification-abundant data and identified 667,034 PSMs. The number of identified PSMs was increased to 693,056 if the scores provided by MODplus were used together as features. Because Percolator did not support the results of MODplus, the comparison was not performed.

**Table 2:** The number of identifications in modification searches

Data	TD	Percolator or FDR_by_MODplus	TIDD
Hela <phospho modification>	152,186	167,925 (+ 10.34%) <Percolator>	172,742 (+ 13.51%)
HEK293 <946 variable modifications>	605,103	653,660 (+ 8.02%) <FDR_by_MODplus>	667,034 (+ 10.23%)

FDR_by_MODplus means the FDR approach provided by MODplus. (Improved % compared to TD)

### Graphical user interface for TIDD

For the convenience of users, we built graphical user interfaces for TIDD using R shiny, which can be downloaded and tested at https://honglan-li.shinyapps.io/project/. It takes database search results in Tab Separated Value (TSV) file format and the corresponding MGF file as input, calculates TIDD features shown in Table 1, and performs the iterative SVM learning. For the SVM learning, users can add (optional) tool-specific features such as match scores provided by the search tools and any user-defined features, and thus decide the optimal feature set of their choice for any search tool.

**Fig. 4:**
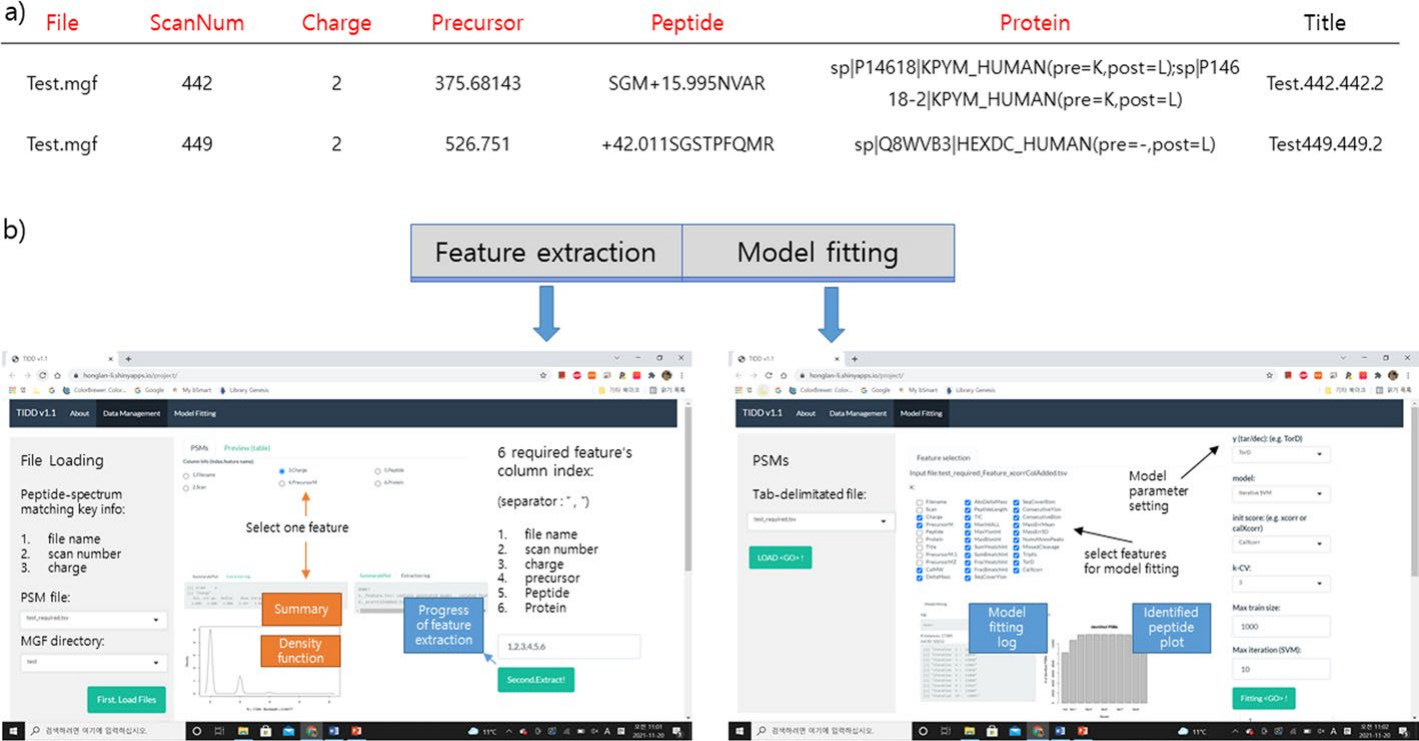
Graphical user interface of TIDD. **a** Example of TIDD input file. **b** Graphical user interface of TIDD

To calculate TIDD features, we take the following 3 types of parameters as input—decoy prefix, fragment tolerance and digestion enzyme. Six numbers representing column indices should be provided so that the numbered column of the input TSV (See Fig. 4a and b) can be used for rescoring regardless of their column heading in the input file. The first three columns should designate “File”, “Scan” and “Charge”, respectively, and they are required to retrieve experimental spectrum from the MGF file specified in the File column. The 4th required column is the mass over charge value of a precursor, named “PrecursorMZ”. The 5th required column is “Peptide”, representing an amino acid sequence together with the possible modification mass information specified as “M + 15.995” (Oxidation at M), for instance (See Fig. 4a). This feature is used to calculate theoretical peptide mass, delta mass, and a series of annotated features. The final feature is “Protein” column, which lists parent proteins with “;” separators, while previous and next amino acids of a peptide sequence are provided as well (e.g., sp|P14618(pre = ‘M’, post = ‘K’), so that we can tell whether the PSM belongs to target or decoy proteins and determine the enzyme specificity at both cleavage sites (fully, semi or none). Users can additionally put a list of column indices corresponding to tool-dependent scores.

After loading files and setting parameters, users can preview their data in “Preview data” section of the application window (in Fig. 4b), then set the SVM parameters and run TIDD. When PSM rescoring is finished, the rescored PSM list, together with their TIDD feature values are generated in the same directory where input PSM results are located.

## Conclusions

TIDD is the first tool-independent and data-dependent PSM rescoring tool, which utilizes the tool-independent universal features regardless of a database search tool adopted and runs iterative SVM learning. TIDD successfully eliminated the requirement for an optimal feature engineering per database search tool, and thus, can be applied directly to any database search results including newly developed ones. We demonstrated the utility of TIDD in validating MSFragger and MODplus search results on 12 human datasets, for which an optimized feature set had not been provided by Percolator. TIDD did not always perform better than Percolator for all the search tools and datasets, which is understandable—it is natural to expect an optimal feature set to give better results for a specific database search tool than a general feature set meant for all search tools. TIDD provides a user interface that allows users to provide arbitrary user-defined features as an input to the initial machine learning stage.

### Availability and requirements

Project name: TIDD (tool-independent and data dependent PSM). Project download page: https://github.com/HanyangBISLab/TIDD.git. Operating system: Platform independent. Programming language: R (v4.1.2 or above), and Java (jdk 17 or above). Other requirements: R packages such as "shiny", "shinythemes", "shinyFiles", "shinydashboard", "e1071", and "ROCR"; License: GNU GPL. Any restrictions to use by non-academics: need.

## Supplementary Information

**Additional file 1.** PSM identification statistics and TIDD feature distributions. It provides the exact number of PSMs identified from various peptide identification approaches, and the distributions of TIDD features on A549 dataset.

## Data Availability

Original data was downloaded from PRIDE (ID: PXD002395, PXD001468). Generated files after database search used in this study are available in https://github.com/HanyangBISLab/TIDD.git.

## Abbreviations

FDR: False discovery rate
GUI: Graphical user interface
ML: Machine learning
MS: Mass spectrometry
MS/MS: Tandem mass spectrometry
PSM: Peptide-spectrum match
SVM: Support-vector machine
TD: Target-decoy approach
