# Supplementary material for: TIDD: tool-independent and data-dependent machine learning for peptide identification
Source: BMC Bioinformatics. 2022 Mar 30;23:109. doi: 10.1186/s12859-022-04640-y (PMC8969291; doi:10.1186/s12859-022-04640-y)
Supplement: Supplementary file 1 — Additional file 1. PSM identification statistics and TIDD feature distributions. It provides the exact number of PSMs identified from various peptide identification approaches, and the distributions of TIDD features on A549 dataset. [file 12859_2022_4640_MOESM1_ESM.pdf]

Table S1. PSM identification results accepted at 1% FDR for Comet search results. Percolator, TIDD, and TIDD with tool-dependent features are all based on iterative SVM learning. The only differences among the three models are the list of features used for SVM learning. TD: target-decoy approach based on e-value. TIDD: tool-independent and data-dependent PSM rescoring. TIDD with tool-dependent features: iterative SVM learning with TIDD features augmented with 4 Comet scores such as "deltacn", "deltacnstar", "SpRank" and logarithm value of "e-value".

	TD	Percolator	TIDD	TIDD with tool-dependent features
A549	104407	111779	111880	112002
HEK293	117753	123131	124594	124641
GAMG	117416	123983	125539	125750
Hela	124812	131193	132512	132654
HepG2	114666	120764	122510	122577
Jurkat	91335	99056	105903	105885
K562	102071	108783	115013	114931
MCF7	107587	116915	122884	123003
RKO	100528	107817	114273	114244
LanCap	91629	100819	107680	107767
U2OS	95972	105352	112250	112247
HEK 293[a]	413367	473093	474232	475577

[a] Large-scale mass spectrometry data studied by Chick, J. M. et al.[1]

Table S2. PSM identification results accepted at 1% FDR for MS-GF+ search results. Percolator, TIDD, and TIDD with tool-dependent features are all based on iterative SVM learning. The only differences among the three models are the list of features used for SVM learning. TD: target-decoy approach based on e-value. TIDD: tool-independent and data-dependent PSM rescoring. TIDD with tool-dependent features: iterative SVM learning with TIDD features augmented with 4 MS-GF+ scores such as "denovo score", "MSGF score", "spectrum e-value" and "e-value".

	TD	Percolator	TIDD	TIDD with tool-dependent features
A549	104857	108535	108670	108692
HEK293	121212	123884	123962	123953
GAMG	117847	122006	122072	122157
Hela	125336	129155	129249	129281
HepG2	114358	118420	118453	118544
Jurkat	93469	102139	101468	101872
K562	106844	113705	112768	112975
MCF7	110316	118908	118179	118662
RKO	102839	110681	110042	110374
LanCap	95511	104259	103401	103823
U2OS	102410	110455	109905	110254
HEK 293[a]	456073	479218	472953	481589

[a] Large-scale mass spectrometry data studied by Chick, J. M. et al.[1]

Table S3. PSM identification results accepted at 1% FDR for MSFragger search results. Percolator, TIDD, and TIDD with tool-dependent features are all based on iterative SVM learning. The only differences among the three models are the list of features used for SVM learning. TD: target-decoy approach based on e-value. TIDD: tool-independent and data-dependent PSM rescoring. TIDD with tool-dependent features: iterative SVM learning with TIDD features augmented with 3 MSFragger scores such as "hyper score", "next score" and "e-value". Iterative SVM using X!Tandem-Percolator features: iterative SVM learning with the feature set used by Percolator on X!Tandem data, while 'deltascore' is missed because MSFragger does not provide this score.

	TD	Percolator	TIDD	TIDD with tool-dependent features	Iterative SVM using X!tanden-Percolator features
A549	86886	88630	102161	102346	99636
HEK293	101662	109420	115710	115820	114511
GAMG	97653	100212	114577	114691	111684
Hela	104691	107313	121472	121636	118896
HepG2	96124	94239	111932	112124	108782
Jurkat	73945	80030	92191	92460	88530
K562	86818	98092	102705	102881	101637
MCF7	86549	92698	106964	107981	103133
RKO	81675	59530	100626	100816	97482
LanCap	73210	80030	93398	94703	89843
U2OS	79901	61375	98136	98333	96321
HEK 293[a]	372231	114344	517209	521190	480876

[a] Large-scale mass spectrometry dataset studied by Chick, J. M. et al.[1]

Figures

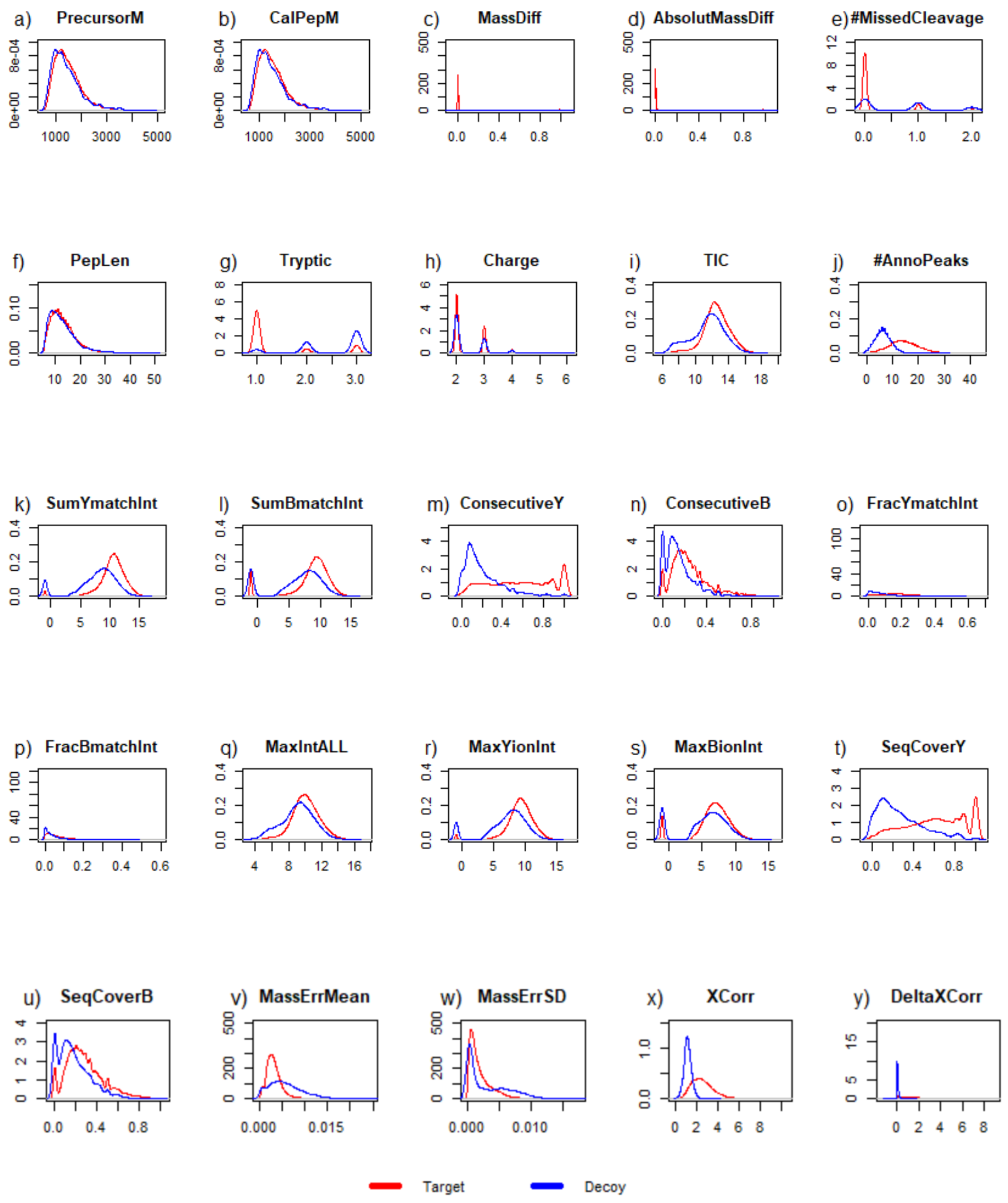

Fig. S1. Data distribution of TIDD features from A549 Comet search results. a) PrecursorM: observed mass of precursor spectra. b) CalPepM: calculated mass of matched peptide. c) MassDiff: mass difference between calculated and observed mass. d) AbsolutMassDiff: the absolute value of the 'MassDiff'. e) #MissedCleavage: number of missed cleavages in the peptide sequence. f) PepLen: the length of stripped peptide sequence. g) Tryptic: vector 0 c-term tryptic; 1 n-term tryptic; 2 fully tryptic. h) Charge: vector from 1 to 6. i) TIC: logarithm value of total ion

current. j) #AnnoPeaks: number of annotated peaks. k) SumYmatchInt: logarithm value of sum of matched y-ion intensity. l) SumBmatchInt: logarithm value of sum of matched b-ion intensity. m) ConsecutiveY: number of consecutively matched y-ions. n) ConsecutiveB: number of consecutively matched b-ions. o) FracYmatchInt: the fraction of 'SumYmatchInt' among 'TIC' p) FragBmatchInt: the fraction of 'SumBmatchInt' among 'TIC' q) MaxIntAll: logarithm value of maximum intensity of spectra. r) MaxYionInt: logarithm value of maximum intensity among matched y-ion. s) MaxBionInt: logarithm value of maximum intensity among matched b-ion. t) SeqCoverY: sequence coverage of y-ion. u) SeqCoverB: sequence coverage of b-ion. v) MassErrMean: mean value of mass difference distribution between experimental and theoretical fragment ions. w) MassErrSD: standard deviation value of the mass distribution between experimental and theoretical fragment ions. x) XCorr: Comet cross correlation score between theoretical and experimental spectrum. y) DeltaXCorr: difference of XCorr score between rank 1 and 2 (if there's rank 2 hit).

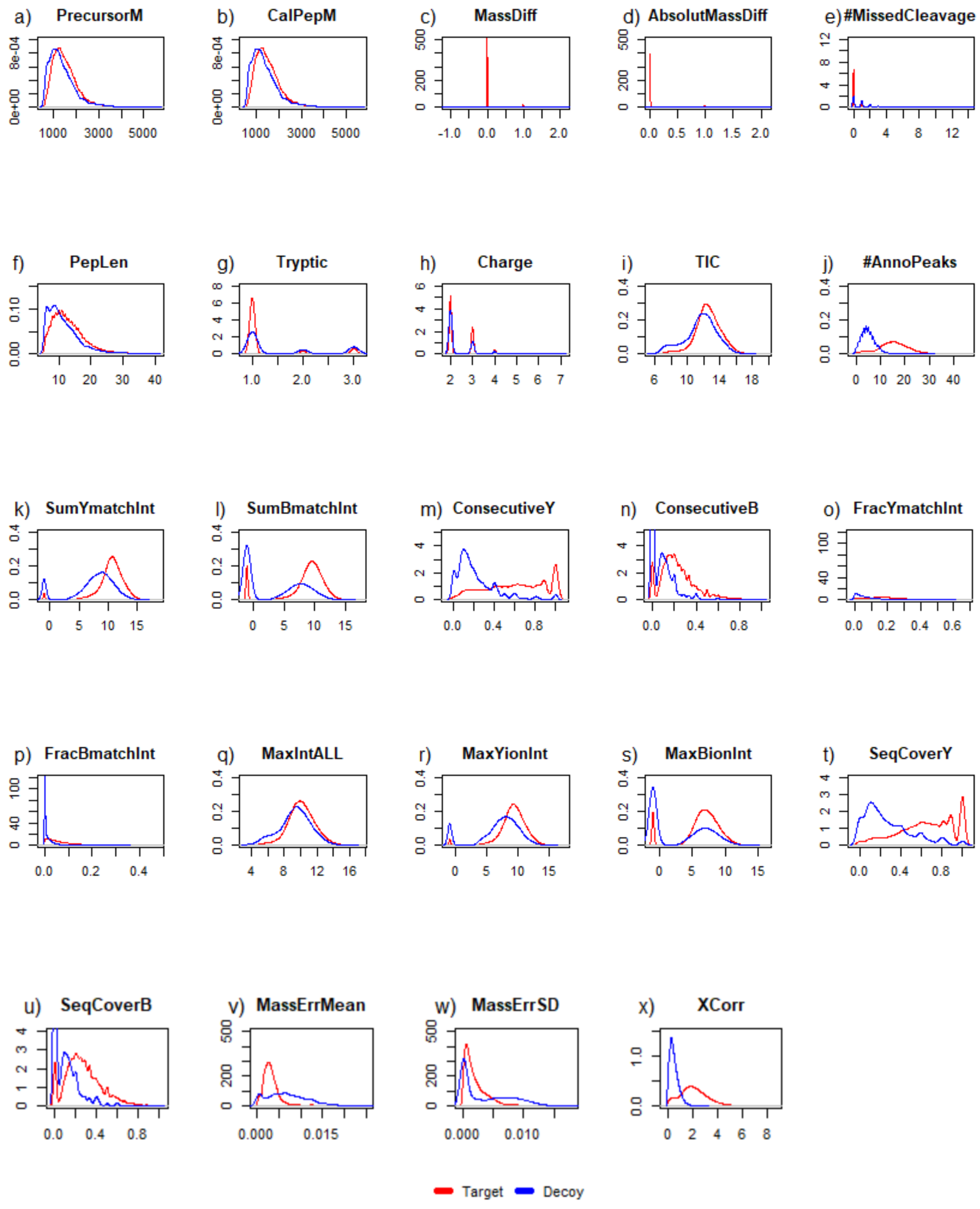

Fig. S2. Data distribution of TIDD features from A549 MS-GF+ search results. a) PrecursorM: observed mass of precursor spectra. b) CalPepM: calculated mass of matched peptide. c) MassDiff: mass difference between calculated and observed mass. d) AbsolutMassDiff: the absolute value of the 'MassDiff'. e) #MissedCleavage: number of missed cleavages in the peptide sequence. f) PepLen: the length of stripped peptide sequence. g) Tryptic: vector 0 c-term tryptic; 1 n-term tryptic; 2 fully tryptic. h) Charge: vector from 1 to 6. i) TIC: logarithm value of total ion current. j) #AnnoPeaks: number of annotated peaks. k) SumYmatchInt: logarithm value of sum of

matched y-ion intensity. l) SumBmatchInt: logarithm value of sum of matched b-ion intensity. m) ConsecutiveY: number of consecutively matched y-ions. n) ConsecutiveB: number of consecutively matched b-ions. o) FracYmatchInt: the fraction of 'SumYmatchInt' among 'TIC' p) FragBmatchInt: the fraction of 'SumBmatchInt' among 'TIC' q) MaxIntAll: logarithm value of maximum intensity of spectra. r) MaxYionInt: logarithm value of maximum intensity among matched y-ion. s) MaxBionInt: logarithm value of maximum intensity among matched b-ion. t) SeqCoverY: sequence coverage of y-ion. u) SeqCoverB: sequence coverage of b-ion. v) MassErrMean: mean value of mass difference distribution between experimental and theoretical fragment ions. w) MassErrSD: standard deviation value of the mass distribution between experimental and theoretical fragment ions. x) XCorr: Comet cross correlation score between theoretical and experimental spectrum.

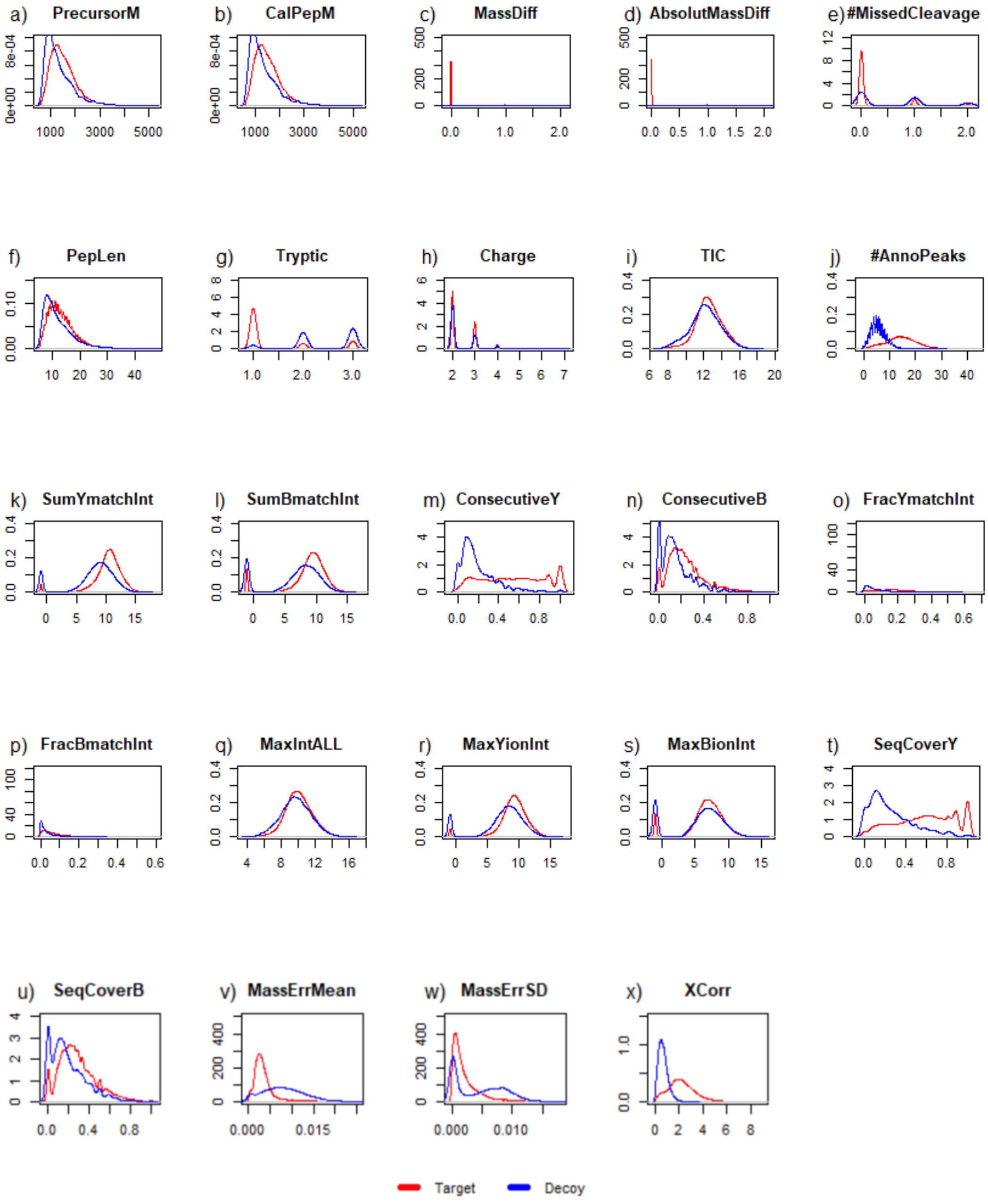

Fig. S3. Data distribution of TIDD features from A549 MSFragger search results. a) PrecursorM: observed mass of precursor spectra. b) CalPepM: calculated mass of matched peptide. c) MassDiff: mass difference between calculated and observed mass. d) AbsolutMassDiff: the absolute value of the 'MassDiff'. e) #MissedCleavage: number of missed cleavages in the peptide sequence. f) PepLen: the length of stripped peptide sequence. g) Tryptic: vector 0 c-term tryptic; 1 n-term tryptic; 2 fully tryptic. h) Charge: vector from 1 to 6. i) TIC: logarithm value of total ion current. j) #AnnoPeaks: number of annotated peaks. k) SumYmatchInt: logarithm value of sum of

matched y-ion intensity. l) SumBmatchInt: logarithm value of sum of matched b-ion intensity. m) ConsecutiveY: number of consecutively matched y-ions. n) ConsecutiveB: number of consecutively matched b-ions. o) FracYmatchInt: the fraction of 'SumYmatchInt' among 'TIC' p) FragBmatchInt: the fraction of 'SumBmatchInt' among 'TIC' q) MaxIntAll: logarithm value of maximum intensity of spectra. r) MaxYionInt: logarithm value of maximum intensity among matched y-ion. s) MaxBionInt: logarithm value of maximum intensity among matched b-ion. t) SeqCoverY: sequence coverage of y-ion. u) SeqCoverB: sequence coverage of b-ion. v) MassErrMean: mean value of mass difference distribution between experimental and theoretical fragment ions. w) MassErrSD: standard deviation value of the mass distribution between experimental and theoretical fragment ions. x) XCorr: Comet cross correlation score between theoretical and experimental spectrum.

Reference

1. Chick, J. M.; Kolippakkam, D.; Nusinow, D. P.; Zhai, B.; Rad, R.; Huttlin, E. L.; Gygi, S. P., A mass-tolerant database search identifies a large proportion of unassigned spectra in shotgun proteomics as modified peptides (vol 33, pg 743, 2015). *Nat Biotechnol* **2015,** *33* (8), 882-882.
